# Gun Violence: A Biopsychosocial Disease

**DOI:** 10.5811/westjem.2018.7.38021

**Published:** 2018-09-10

**Authors:** Stephen W. Hargarten, E. Brooke Lerner, Marc Gorelick, Karen Brasel, Terri deRoon-Cassini, Sara Kohlbeck

**Affiliations:** * Medical College of Wisconsin, Department of Emergency Medicine, Milwaukee, Wisconsin; † Children’s Hospital and Clinics of Minnesota, Minneapolis, Minnesota; ‡ Oregon Health and Science University, Department of Surgery, Portland, Oregon; § Medical College of Wisconsin, Department of Surgery, Milwaukee, Wisconsin; ¶ Medical College of Wisconsin, Comprehensive Injury Center, Milwaukee, Wisconsin

## Abstract

Gun violence is a complex biopsychosocial disease and as such, requires a multidisciplinary approach to understanding and treatment. Framing gun violence as a disease places it firmly within medical and public health practice. By applying the disease model to gun violence, it is possible to explore the host, agent, and environment in which gun violence occurs, and to identify risk factors to target for prevention. This approach also provides an opportunity to address scientifically inaccurate assumptions about gun violence. In addition, there are many opportunities for medical communities to treat gun violence as a disease by considering and treating the biologic, behavioral, and social aspects of this disease. The medical community must answer recent calls to engage in gun violence prevention, and employing this model of gun violence as a biopsychosocial disease provides a framework for engagement.

Gun violence is a pervasive public health burden in the United States. Annually, over 36,000 Americans die from firearm-related events; tens of thousands are injured.^1^ The medical community has periodically called for framing gun violence as a public health/medical issue.^2^–^9^ Given the impact of gun violence on health and longevity,^10^ others have suggested that physicians have a moral obligation to address gun violence.^11^,^12^ More recently, others have called upon physicians to integrate firearm-related education about safety with their patients.^13^

Calls for engagement have increased with multiple physician organizations calling for action.^2^,^14^ In much the same way that human immunodeficiency virus (HIV) rates grew unchecked until we began to acknowledge that it was a biopsychosocial disease that could be prevented and controlled, and scientifically we moved past the social stigmas of a disease first recognized as largely affecting homosexual men, gun violence will continue unchecked until we invest in research to discover effective means to reduce it. To fully engage physicians and other sectors of the healthcare community, we need to frame gun violence as a biopsychosocial disease.^12^ We know that gun violence follows predictable patterns just like infectious diseases and other illnesses.^15^ For example, young African-American males are at increased risk of firearm-related homicide, while older White males are at increased risk for firearm-related suicide. Through an understanding of the risk factors for a disease, we can identify means of control and prevention.

The disease model approach was first advanced in the 19th century and continues today. With a science driven understanding of disease etiology, physicians and other civic leaders were positioned to discover vaccines, thus changing the environments that breed the vectors of illnesses, while identifying high-risk groups for preventative interventions– all driven by the science of discovery. We are seeing this unfold today with the Zika virus,^16^ and the prevention strategies of other communicable diseases such as tuberculosis and HIV that continue to benefit from the rigorous application of the disease model. By identifying and understanding the disease agent, its vector of transmission, and the high-risk hosts and environments, all sectors of civil society – healthcare, public health, businesses, schools, fire and police agencies– can work in concert to institute interventions that reduce morbidity and mortality. These interventions may prevent exposure to the agent that causes disease, reduce the chance of becoming ill if exposed, or limit the damage after the disease is contracted.

Scientific investigations have advanced the disease model to include other causes of cellular/organ damage from a variety of etiologic agents.^17^ For decades, clinicians and public health professionals have been trained to understand the definition of disease as having four components: etiology, pathogenesis, morphologic changes, and clinical significance.^17^ We have learned that the etiologic agents of diseases are categorized into biologic and physical agents that interact with cells and organs, resulting in disruptions of cell walls and the release of substances that cause additional destruction.^18^ For example, with the Ebola virus disease, the pathogenesis occurs over days and can manifest up to 21 days after exposure. The virus begins to replicate and results in morphologic changes in cells/organs that manifest as a constellation of symptoms, resulting in nausea, vomiting, and diarrhea, leading to dehydration, organ failure and death.

Analogously, the kinetic energy from a bullet is the physical agent of gun violence. The kinetic energy imparted by the speeding mass of the bullet results in the tearing of cellular membranes, leading to edema, fractures, and bleeding, resulting in organ failure, shock, and death. The energy (KE=1/2MV^2^), is transmitted to the host/patient from the bullet – penetrating the skin, entering the body, and transmitting the energy, leading to temporary and permanent cavity formation, and a sterile injury to the patient.^18^,^19^ The pathophysiology of this disease has received limited examination because the agent (kinetic energy) causes destruction so quickly (less than 0.1 sec).^20^ The high-speed video camera is the “microscope” for this rapidly occurring disease. It is through this “lens” that we can document the temporary and permanent cavity formation that is the hallmark of the biology of this disease.^19^–^21^ This dramatically brief pathophysiology limits acute interventions during the release of kinetic energy and is distinctive since diseases from other agents, such as viruses and bacteria, clinically develop over days or weeks.

By framing gun violence as a biopsychosocial disease,^22^ it engages the healthcare community of physicians and nurses, complements the necessary multidisciplinary approach to advance our scientific understanding, and informs host, agent/vector, and environmentally-focused interventions beyond the immediate biology of fractures, bleeding, and edema. This is critically important since preventing and controlling gun violence will not occur to any significant degree until we begin to approach it in a manner similar to controlling other biopsychosocial diseases such as HIV. One immediate benefit of framing gun violence as a disease is the opportunity to address misleading/limiting statements as scientifically inaccurate, yet repeated over and over again. One of the most common of these is: “Guns don’t kill people, people kill people.”

The disease model provides us with accuracy: the bullet and its kinetic energy shreds, tears and destroys cells, and damages organs, leading to death and disability. While the behavioral health issues that result in a person pulling a trigger and releasing the energy need to be better understood, first and foremost we need scientifically accurate statements that advance the necessary, challenging discussions. By recognizing that bullets kill people, the gun, which carries the bullets, becomes a necessary focus of intervention. One such strategy would be to limit the rate of the release of bullets by, for example, banning bump stocks or automatic weapons, or by reducing the amount of potential energy the gun can carry (magazine capacity). Without this framing we will be limited to education of our patients^13^ or continue to be stuck, mired in debates that do not advance scientific understanding, but only entrench positions. We limit progress related to gun violence by not addressing the environment and the social context and psychological antecedents and outcomes of this disease that affect patients, families and communities.^23^,^24^

In addition to the injury caused by a bullet, the body’s own biologic stress response is activated and involves a cascade of bodily systems, including stress hormones. While this biological response is adaptive, sustained activation of the acute stress response degrades healthy adaptation following a life-threatening situation. This is even further exacerbated when an individual experiences psychological stress after trauma, particularly post-traumatic stress disorder (PTSD). The social context of gunshot-wound patients is paramount, including the community/neighborhood the survivor is coming from, the location of the wounding event, and the environment to which they have no choice but to return. Unfortunately, issues such as familial retaliation and the maintenance of perceived strength within communities with high levels of violence can perpetuate the cycle of gun violence, “spreading” the risk of the disease. Social, environmental, physical, and psychological pre-, peri-, and post-injury factors influence the course of gun violence as a disease and therefore should be treated from this biopsychosocial perspective.

There are many opportunities for medical communities to treat gun violence as a biopsychosocial disease. Increasingly, trauma centers^25^ are recruiting clinical psychologists to provide behavioral health interventions that complement the surgical team’s emphasis on the biology. While the integration of behavioral health specialists is occurring within centers where the disease is most likely to be treated, the majority of centers are not yet advancing care with this integrated approach. Behavioral/social interventions include hospital-based, violence-prevention programs, where the focus is to address the social and behavioral issues of gun violence and to prevent recidivism. In some instances, primary care physicians are^26^ trained in assessing exposure to trauma to understand the social context of the patient’s health. They can provide recommendations for psychological care if distress is evident. While these examples exist within healthcare, unfortunately they are not the norm. To move disease prevention forward, significant development of integrated multidisciplinary programs is needed. Additionally, more research is needed in the inpatient setting of trauma centers to better understand the psychosocial elements of this disease to maximize outcomes and reduce recidivism.

The importance of this framing distinction can be more easily seen when we consider prior and ongoing work to reduce the burden of acute injury from car crashes. We have achieved considerable success in the application of the disease model, which has resulted in significant reductions in death and disability over the past 50 years.

Evidence-based policies such as seatbelt laws and significantly improved car and road designs that attenuate and control the energy exchange with passengers and drivers – all components of the disease model – have been systematically investigated and advanced.^29^

In the first 10 years of the 21st century there were substantial declines in morbidity and mortality from other public health burdens such as vaccine-preventable diseases, childhood lead poisoning, cardiovascular disease, workplace-associated injuries, and cancer, while improvements were made in areas such as maternal and fetal health.^27^ However, similar improvements have not been made in firearm deaths during this time; in fact, deaths from firearms continue to rise. This may be attributed, at least in part, to the relative paucity of funding for firearm-violence research, due in part to the 1996 Dickey amendment, which states that, “None of the funds made available for injury prevention and control at the Centers for Disease Control and Prevention may be used to advocate or promote gun control.”^28^

As a society, we have achieved success in controlling infectious diseases with a focused, disease-model approach, and we have successfully expanded the use of the disease model to prevent and control non-communicable diseases such as cancer and heart disease. We have used this approach for other challenging biopsychosocial disease burdens such as smoking and alcohol abuse.^30^ Further, it was only once we blunted the political stigma stunting our progress in combating HIV that the most significant discoveries took place and lives were saved. Yet we have not taken the next step in using the disease model to prevent and control gun violence, in part due to the relative lack of funding, and therefore the relative lack of investigation. Framing gun violence as a disease places it firmly within medical and public health practice. Interventions across multiple sectors, informed by comprehensive, linked data and rigorous, adequately-funded research, can be used to prevent injuries, improve acute care and rehabilitation, and inform and evaluate program and policy interventions. These can ultimately reduce morbidity and mortality.

This framing opens up important areas of research and prevention strategies that can and must be organized to address all aspects of the disease: high-risk youth; adults and elderly; the gun and the bullets; and the environment.^30^ Specific examination of the gun and its design/safety characteristics open up areas of potential interventions. Much like reducing a child’s access to the energy contained in a medicine container resulted in decreases in unintentional chemical injury from aspirin and Tylenol,^31^ banning bump stocks would reduce the rate of energy release that was so tragically seen in the Las Vegas shooting of October 2017. Designing a “smart” gun, which leverages new technologies to identify a gun’s owner and prevent its use by others, could also have the potential to reduce the number of accidental (unintentional) deaths and suicides.^33^, ^34^ In this environment, requiring background checks on all gun sales has the potential to further reduce unauthorized access.^35^

Recent calls to engage the physician and public health communities in addressing gun violence^6^,^11^^,36^ must be answered by the medical community. Kaiser Permanente, one of largest health systems in the U.S., has recently approved a $2 million expenditure to study gun violence prevention.^37^ By framing gun violence as a biopsychosocial disease we can move beyond acrimony and fear, use the tools that have been honed over centuries to advance science, and prevent and control this disease burden that adversely impacts our patients, families, and communities across the U.S. and the world.
